# Magnetic Characterization of the Infinitene Molecule

**DOI:** 10.1021/acs.jpca.2c02339

**Published:** 2022-06-06

**Authors:** Guglielmo Monaco, Riccardo Zanasi, Francesco F. Summa

**Affiliations:** Department of Chemistry and Biology “A. Zambelli”, Università degli Studi di Salerno, via Giovanni Paolo II 132, Fisciano 84084, Salerno, Italy

## Abstract

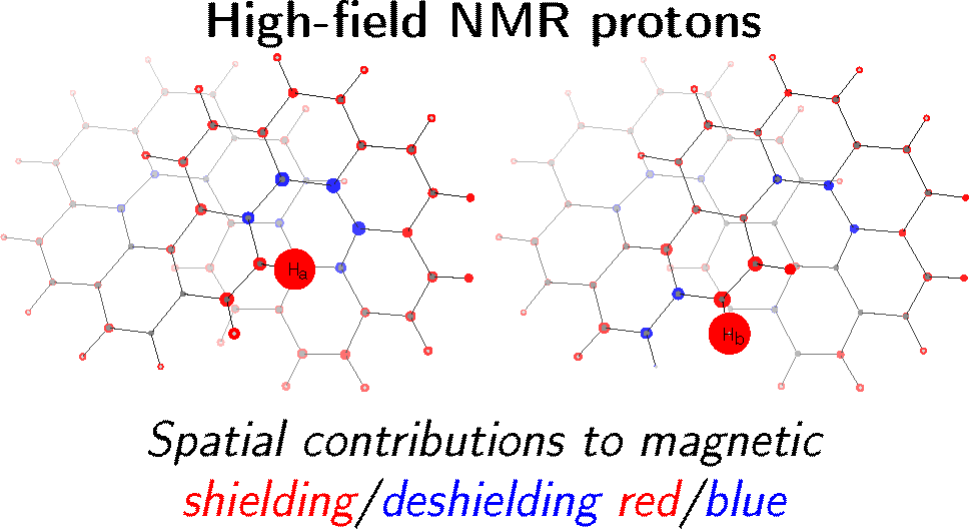

The origin-independent current density induced by a perpendicular magnetic field in the infinitene molecule has been calculated, confirming the recently presented result by Orozco-Ic et al. (Phys. Chem. Chem. Phys.2022, 24, 6404−64093526214810.1039/d2cp00637e) of two disjointed global current pathways along the edges formed by 24 carbon atoms having the form of the infinity symbol. The current strength has been assessed along the C–C bonds forming the two separate circuits, whose particular shape provides a diamagnetic exaltation which is only 73% of the expected value for this aromatic molecule. Through space currents have been found along the bond paths determined by the electron density gradient, whose strength is 10% that of the aromatic benzene ring current. It is shown that the pair of high-field ^1^H NMR experimental signals carry the signature of the two global currents, which are counterrotating inside the fjord regions with respect to the rim of the coronene subunits.

## Introduction

The large family of polycyclic aromatic hydrocarbons (PAHs) has recently acquired a new member referred to as “infinitene” due to its helically twisted structure resembling that of the infinity symbol (∞).^1^ Even at first glance, infinitene looks to be a truly fascinating molecule, in particular with regard to some issues concerning the nature of its aromaticity in terms of the magnetic criterion.^2^ In fact, besides enhanced stability, specific reactivity, and bond equalization, it is well recognized that the magnetically induced current density is intimately linked with the aromaticity concept and it is an essential ingredient for the interpretation of the magnetic response of conjugated π-systems, such as the nuclear magnetic shielding in NMR spectroscopy and the exaltation of diamagnetism.^3,4^ However, despite recent progress,^5,6^ the inference of the actual shape of the ring current in PAHs, starting from ^1^H NMR data or the calculations of a few nucleus-independent chemical shifts (NICSs),^7^ is not straightforward, and this is especially true for curved PAHs, such as infinitene.

Infinitene can be seen as two cleaved coronene ([6]circulene) subunits, twisted as two homochiral helices and stitched together by both their ends, in such a way that the rim of one coronene is attached to the hub of the other, forming two circuits of equal length containing 24 carbon atoms each. For coronene it is known that two counterrotating ring currents are induced by a perpendicular magnetic field, one strongly diatropic on the rim and the other weakly paratropic on the hub, which provide evidence for a resultant global aromaticity of the molecule.^8^ Therefore, a number of questions concerning the shape and strength of ring currents (if any) arise:

(i) What pathways do the currents travel through?
(ii) Are they global, or local to Clar sextets?
(iii) Which tropicity do they display?
(iv) How do their strengths compare with the benzene ring current?
(v) How large is the exaltation of diamagnetism for this aromatic molecule?
(vi) How can the high-field ^1^H NMR signals be justified on the basis of the actual current tropicity?

Nowadays, there exist powerful methods that can be readily used to solve the problem by calculating directly the magnetically induced current density for any orientation of the inducing magnetic field.^9,10^ Therefore, not at all surprisingly, although the work of synthesis was very recent,^1^ a theoretical paper elucidating the current pathways in infinitene has already appeared^11^ when our article was still in progress. In that work it has been clearly shown that the induced current is characterized by two aromatic, nonintersecting global π-electron current pathways, formed by the two circuits of 24 carbon atoms along the edges shaped as the infinite symbol. This finding answers the first three of the above questions. In addition, it addresses the question of whether the molecule follows Hückel 4*n* + 2 or Möbius 4*n* aromaticity rules, showing that infinitene does not belong to any of these classes of molecules. Nonetheless, the last three questions remain. It is the purpose of this article to elucidate these additional aspects.

## Methods

**Figure 1:**
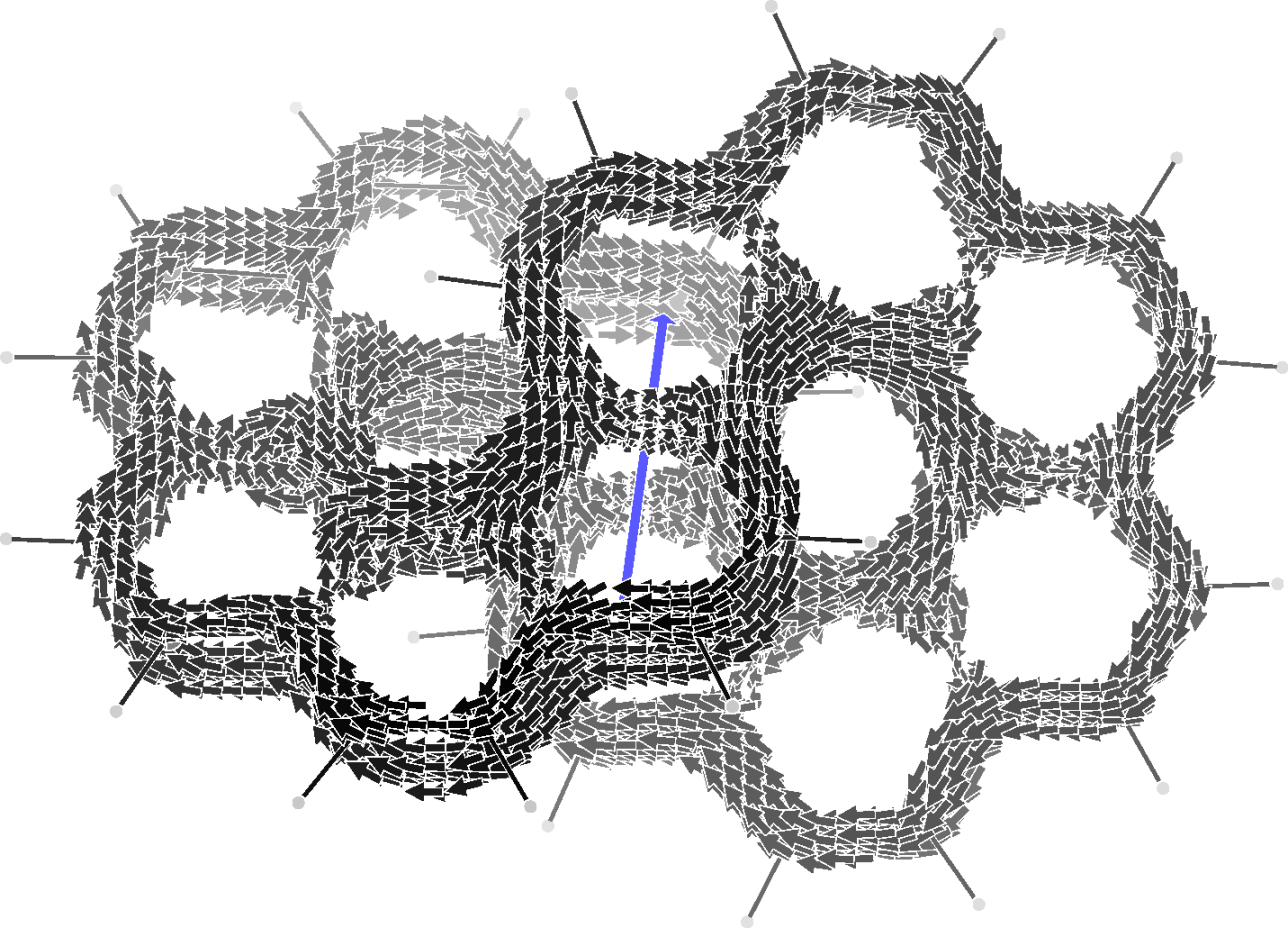
π-Electron current density induced in infinitene by a unitary magnetic field (blue arrow) parallel to the Cartesian *x*-axis, corresponding to *C*_2_^*x*^ symmetry element. The other two binary axes of *D*_2_ must be combined with the time reversal operator. Currents lower/higher than 0.05/0.1 au are not shown.

We have taken the geometry of the (*P*,*P*)-isomer of infinitene, optimized at the PBE0/6-311+G(d,p) level of theory in the gas phase, reported by Krzeszewski et al.^1^ The symmetry point group of the structure is *D*_2_ with the Cartesian *x*-axis perpendicular to the central C–C bonds of the stacked naphthalene subunits, as shown in Figure 1. Then, we have performed the calculation of the magnetically induced current density using the CTOCD-DZ method to ensure origin-independent results,^12^ adopting the B97-2 (ref (13))/6-311+G(2d,p) (ref (14)) level of theory in the gas phase. The Gaussian 16 program^15^ was used to obtain the perturbed molecular orbitals with the CSGT^16^ keyword and the SYSMOIC program package^10^ to perform the actual calculation of the current density. The entire procedure is a very simple one; details can be obtained visiting the link reported in ref (10).

## Results and Discussion

In infinitene σ/π orbital separation is not strictly possible. However, descendants of p orbitals can be easily detected by using a combination of symmetry arguments and by inspection. The 156 doubly occupied molecular orbitals of *D*_2_ infinitene can be partitioned into 48 1s cores plus 84 σ orbitals, spanning the symmetries
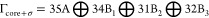
1and 24 more orbitals that can be tentatively classified as π orbitals
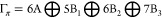
2

Using some facilities contained in the SYSMOIC package, we have readily identified the set composed by HOMO, HOMO – 1, HOMO – 2, ..., HOMO – 14 plus HOMO – 17 as nearly true π orbitals, as confirmed by inspection with GaussView. Searching for the remaining eight orbitals was revealed to be unsuccessful due to large σ/π mixing. Then, only HOMO, ..., HOMO – 14 plus HOMO – 17 have been used in the calculation of the current density map induced in the π-electron cloud. The calculated CTOCD-DZ π-electron current density, induced by a unitary magnetic field parallel to the Cartesian *x*-axis, is shown in Figure 1. Considering that the typical benzene ring current has a maximum value of about 0.08 au,^17^ we have applied a lower/higher cutoff of 0.05/0.1 au to the current density values calculated over a grid of 12 × 20 × 28 *a*_0_ in steps of 0.4 *a*_0_. As can be observed, the result is impressively clear and in full agreement with the current pathways reported in ref (11). Two distinct current flows can be seen to occur along the equivalent circuits of 24 carbon atoms, each one formed by all the K-regions of a coronene subunit plus the internal fjord region of the second coronene subunit. Looking carefully, the two global ring currents are really disjointed, as already noted, since along the radial bond of the coronene subunits the current goes in opposite directions. As can be observed, the homotropicity of the current pathways is a direct consequence of the magnetic group of symmetry,^18^ which can be worked out according to Tavger and Zaitsev.^19^ Actually, when the magnetic field is parallel to *C*_2_^*x*^, the magnetic group is *D*_2_(*C*_2_^*x*^) = (*EC*_2_^*x*^*RC*_2_^*y*^*RC*_2_^*z*^), where *R* is the time reversal operator. Every symmetry element can be easily seen looking at the current density field depicted in Figure 1.

**Figure 2:**
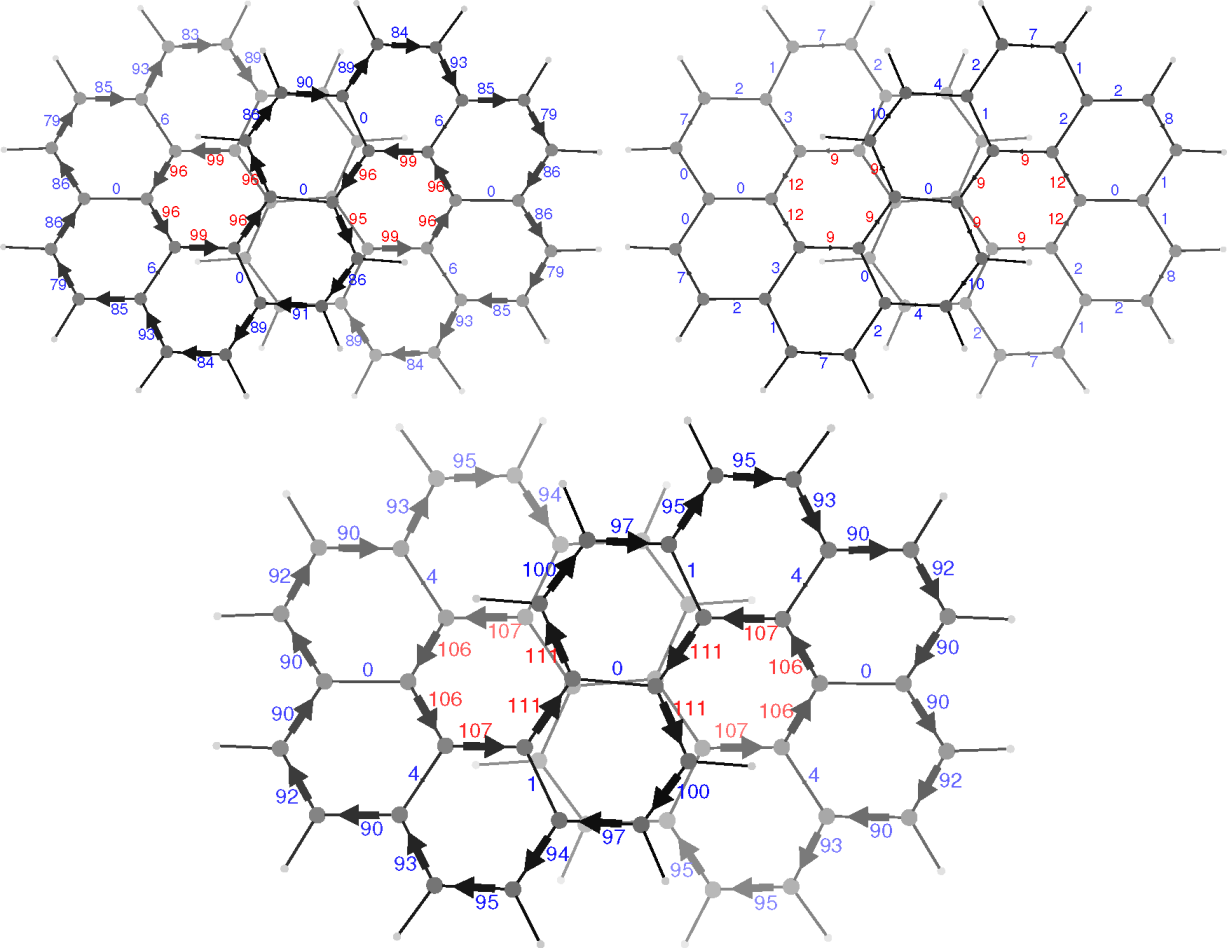
Calculated bond current strengths in infinitene. Top left, π-electron contribution from the orbital set used to compute Figure 1; top right, contribution from all the remaining orbitals; bottom, all electrons. See caption of Figure 1 for the orientation of the inducing magnetic field. Numbers attached to each arrow give the current strength in percentage with respect to the benzene ring current strength of 12.0 nA/T taken as reference.^21^

**Figure 3:**
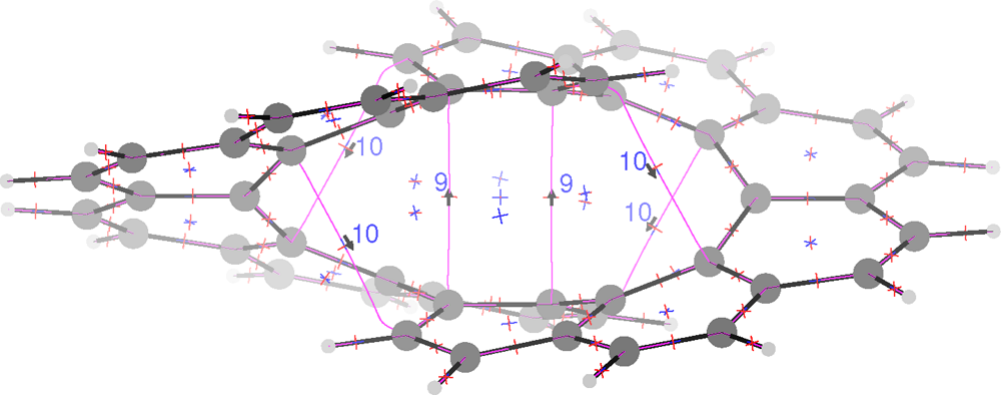
Molecular graph of the electron density gradient. Bond critical points, i.e., (3,–1) saddle points of the ∇ρ vector field, are marked with crosses having two red arms and one blue arm; ring critical points, i.e., (3,+1) saddle points, are marked with crosses having one red arm and two blue arms. Bond paths are shown in magenta. Direction of the current and current strengths are computed for the magnetic field in the *x* direction.

This qualitative result is confirmed by the quantitative representation given by the calculated all-electron bond current strengths^20^ reported in the bottom panel of Figure 2. The two panels in the top of Figure 2 have been calculated separating the contribution given by the set of 16 orbitals identified as nearly true π orbitals (top left) and the contribution given by all the remaining 140 orbitals (top right), which includes also the eight orbitals showing large σ/π mixing. What is shown in the top right panel of Figure 2 can be due to delocalized currents coming from σ electrons, which are known to be somewhat less than 10% of the total current in benzene,^22^ but also due to the residual σ/π mixing. At any rate, in the bottom panel of Figure 2 a real representation is given of the current delocalization, which is not affected by any ambiguous assumption. As can be observed, the current strength along the terminal K-regions is about 92% of the benzene current strength; it increases in the intermediate K-regions and reaches a maximum of 111% within the fjord regions. The current strength for the radial bonds of the coronene subunits is vanishing for all those bonds which are interchanged by *C*_2_ symmetry elements and is not larger than 0.5 nA/T in all other cases. This shows quantitatively the disconnection of the two global ring currents. The apparent lack of current conservation is due to currents which do not follow the bond skeleton, but go through space, as already reported in ref (11). In particular, we have found that these through-space currents follow the trajectories of the molecular graph that is obtained by the topological analysis of the electron density gradient,^23^ shown in Figure 3. As can be observed, six bond critical points (BCPs) plus seven ring critical points (RCPs) are detected within the space between the two helices. BCPs are found exactly midway each pair of unbounded carbon atoms having the shorter distances, i.e., the two pairs formed by the central carbon atoms of the stacked naphthalene subunits, which are at 2.954 Å far apart, and the other four around these central pairs as shown in Figure 3, for which the C–C distance is 2.978 Å. Similar bond paths have been previously reported for helicenes.^24^ The two carbon atoms of each pair are connected by a bond path (shown as magenta lines) corresponding to the trajectories of ∇ρ, leaving the BCP along the direction of the positive eigenvector of the Hessian ∇∇ρ calculated in the BCP. Using the same procedure used to calculate the bond current strengths along the bond skeleton, we have determined the strengths of the current induced by a magnetic field parallel to the *x*-axis for the bond paths shown as magenta lines within the two helices. In this way we estimate a through-space current strength of 1.14 nA/T (9% of the benzene ring current) for the central bond paths and 1.17 nA/T (10%) for the oblique bond paths. Remarkably, these through-space currents stem from the nonclassical component of the current density, as they are almost parallel/antiparallel to the inducing field. Most important for the current conservation, these current strengths are found to go in opposite directions and supply the missing current in Figure 2.

Owing to the low symmetry of infinitene, the analysis of the orbital symmetries^25^ provides only a little further understanding. As usual, frontier orbitals provide the major contributions, i.e., the 16 π orbitals considered above furnish nearly the total current density, but almost all of them give rise to transitions to low-lying virtual orbitals that are active for both translation and rotation. For example, the symmetries of the LUMO, LUMO + 1, and LUMO + 2 turn out to be B_2_, B_1_, and A, respectively, while the symmetries of the HOMO, HOMO – 1, HOMO – 2, and HOMO – 3 are B_3_, A, B_3_, and B_1_; a full set of transitions active for both translation and rotation arise from these few orbitals.

**Figure 4:**
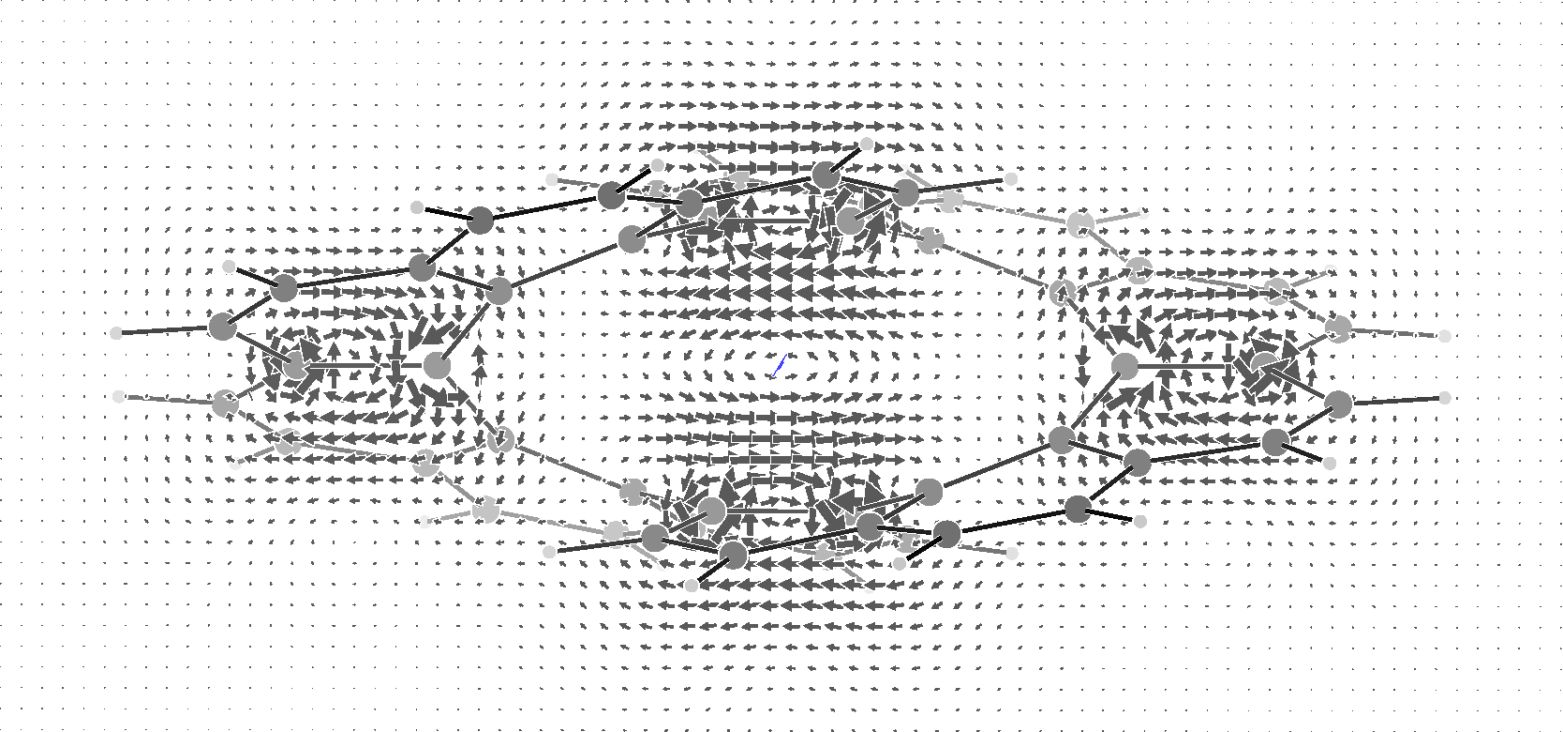
π-Electron current density induced in infinitene by a unitary magnetic field parallel to the Cartesian *y*-axis over a plane containing the four central carbon atoms. Paratropic current is counterclockwise.

For planar aromatics, the perpendicular direction of the perturbing magnetic field is the most studied. However, in the case of infinitene a brief argument can be added for the current density induced by a magnetic field perpendicular to the central pseudocyclobutadiene ring shown in Figure 3, since a paratropic (antiaromatic) current is expected to occur within this central region of the molecule for a magnetic field parallel to the *y*-axis, in this case. The result of the calculation is shown in Figure 4, where the expected paratropic current can be observed in all its beauty.

### Magnetizability

Coming back to the perturbation along the *x*-axis, seen from above, each current loop seems to include a diatropic portion and a paratropic portion, which coincide with the diatropic circulation on the rim of a coronene subunit and the paratropic circulation on the hub of the other subunit; see in Figure 2 blue/red numbers for diatropic/paratropic. Notably, the current strength diatropic/paratropic ratio is reversed with respect to pristine coronene, where the current strength on the rim is 3 times larger than that on the hub. This has a consequence on the computed magnetizability, whose component ξ_*xx*_ in infinitene is −812 cgs ppm (continuous set of gauge transformations (CSGT) method), which is much less than 2 times ξ_∥_ = −586 cgs ppm in coronene (same method). Let us expand the topic in a more formal way, assuming the conventional correlation that links linearly the diamagnetic exaltation Λ to the magnetic anisotropy Δξ.^26^ According to such a convention, the bigger |Δξ| is, the bigger Λ will be. Molecular magnetizability components for the main principal axes *K*_3_, *K*_1_, and *K*_2_ are collected in Table 1, along with those of the reference compounds coronene and benzene, calculated by using the same CSGT/B97-2/6-311+G(2d,1p) method for all molecules. Here, *K*_3_ is used to indicate the component parallel to the main symmetry axis in benzene and coronene, while it indicates the component parallel to the *C*_2_^*x*^ symmetry axis of infinitene. Then, the anisotropy is calculated as
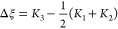
3

**Table 1:** Computed Molecular Magnetizabilities ξ in cgs ppm (CSGT Method)^a^

molecule	*K*_3_	*K*_1_	*K*_2_	ξ_Av_	Δξ	Δξ/Δξ_benzene_
infinitene	–812.3	–245.1	–215.4	–424.3	–582.1	8.8 < 12
coronene	–586.1	–103.2	–103.2	–264.2	–483.0	7.3 > 6
benzene	–99.4	–33.4	–33.4	–55.4	–66.0	1

a *K*_3_ is the largest component of magnetizability in absolute value, conventionally the parallel components, *K*_1_ and *K*_2_, are perpendicular components. Δξ is the anisotropy.

Remarkably, it turns out that the anisotropy of infinitene is fairly less than those expected for an aromatic compound. Indeed, in comparison to coronene, for which Δξ is calculated to be more than 7 times the anisotropy of benzene (in this case the rim current strength is remarkably high), infinitene shows a Δξ that is only 8.8 times the benzene anisotropy. This result is easily understood on the basis of the current density strengths reported in Figure 2; i.e., the two paratropic islands reduce Λ in relation to their surface. Roughly speaking, one can consider 12 times the benzene anisotropy as expected for the anisotropy of infinitene, being 12 the number of benzene rings forming the molecule, minus 2 paratropic rings for the fjord regions, minus some more effects due to nonplanarity, to arrive at the 8.8 ratio. In these terms we estimate a Λ reduced to 73% of the value expected for a planar stripe of benzene molecules.

### ^1^H NMR Chemical Shifts

The experimental ^1^H NMR spectrum of infinitene presents six doublet peaks within the aromatic region (from 6.4 to 8.2 ppm) which have been successfully assigned to the various kinds of protons on the basis of a very good comparison with calculated chemical shifts at the GIAO-DFT/B3LYP/6-311+G(2d,p) level of theory in CHCl_3_ with an SMD solvent model.^1^ It was suggested that it is reasonable to attribute the larger shielding of the protons attached to the central naphthalene (H_a_ and H_b_, same labeling as in ref (1)) to an effect from the ring current on a lower benzene ring. No other argument was given supporting this idea. Given the particular form of the magnetically induced current density, i.e., there are no ring currents localized on benzene rings, we have considered that a different explanation could exist.

To see that, we have computed the spatial contributions to the ^1^H NMR magnetic shieldings, at the CSGT/B97-2/6-311+G(2d,1p) level, using the method proposed by Jinger et al.^27^ For the application of this method, integration of the magnetic shielding density function^28^ has to be performed adopting Becke’s partition scheme for the calculation of molecular integrals.^29^ Due to the fairly high sensitivity of the atomic contributions on the atomic size adjustments chosen to decompose the molecular space,^30^ the BCP positions of the electron density gradient^23^ have been used to define the heteronuclear cutoff profiles.^31^

In planar aromatics the out-of-plane component of the nuclear magnetic shielding tensor is the one of major interest. Owing to the disjointed global current density induced by a magnetic field parallel to the *x* direction, as discussed above, a similar importance is expected to occur for the *xx* component of the tensor. Therefore, we have focused our attention on both the *xx* and isotropic components of the proton magnetic shieldings.

**Figure 5:**
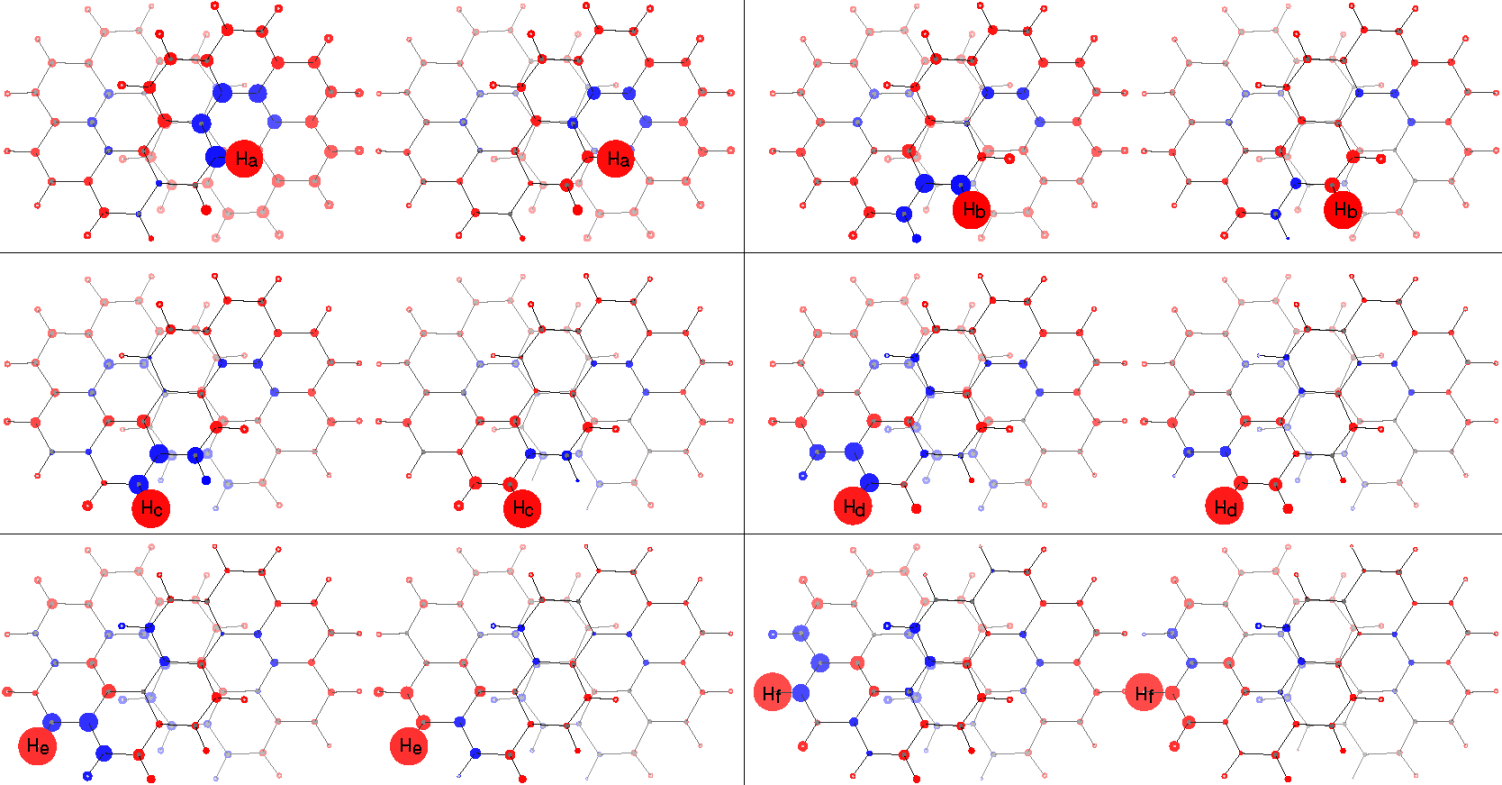
Spatial contributions to the proton magnetic shieldings. See text for details.

Results are assembled in Figure 5 formed by six panels, one for each symmetry unique proton of the molecule (same labeling as in ref (1)). In each panel, contributions to the *xx* component of the tensor are plotted on the left beside the contributions to the isotropic component (tensor average value) on the right. Spatial contributions are represented as spheres of radii proportional to the cubic roots of the calculated values and centered on the corresponding nuclei. Shielding/deshielding contributions are shown in red/blue.

According to the Biot–Savart law^32^ the current effect decreases with the square of the distance. Moreover, closed current loops around atomic nuclei or centered on chemical bonds that do not cover the reference position provide negligible effects irrespective of their strength. Following this key of interpretation, common to all protons it can be seen that

(i) A main shielding contribution (core contribution) is given by the molecular region around each proton.

(ii) The nearest atoms provide the next important contributions.

(iii) Sizable contributions come from atoms at intermediate distances, or even far away, as a consequence of the globally delocalized current.

(iv) The contribution given by the bonded carbon atom to the *xx* component of the magnetic shielding is always negative (deshielding), which turns always positive (shielding) for the isotropic component.

(v) Large deshielding contributions from the next-nearest atoms are negative to both *xx* and isotropic components.

(vi) With the exception of the attached carbon atom and some very minor ones, the isotropic contributions closely resemble the *xx* contributions, revealing the dominant role played by the delocalized current, even if moderated to some extent by the perpendicular components.

Going into details, it can be observed that all pictures for protons H_c_, H_d_, H_e_, and H_f_ (see middle and bottom rows of Figure 5) show similar features. For H_b_ there are larger contributions coming from lower benzene rings. For H_a_ the picture is completely different, showing important deshielding contributions from the carbon atoms forming the fjord region plus a crown of shielding contributions all along the rim of the coronene subunit in which the proton is inserted.

To deal with such a tangled situation, we have collected in Table 2 the core contribution and the sum of all shielding (positive) and deshielding (negative) spatial contributions to the isotropic component of the magnetic shielding of the infinitene protons. Total values have also been transformed in calculated chemical shifts δ_cal_ relative to TMS using^33^
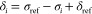
4where σ_ref_ is the computed shielding constant for the same nucleus in a reference compound, σ_*i*_ is the computed shielding constant for the nucleus in the molecule of interest, and δ_ref_ is the experimental chemical shift for the reference compound relative to TMS. For aromatic protons we use C_6_H_6_ as the reference compound, adopting δ_ref_ = 7.36 ppm in CDCl_3_.^34^ First of all, we remark the good comparison with experimental chemical shifts:^1^ the order of the signals is correctly computed and the largest deviation is only 0.15 ppm for H_a_.

**Table 2:** Contributions to the Isotropic Component of the Proton Magnetic Shieldings: Core, Sum of Shielding Spatial Contributions (SSSC), Sum of Deshielding Spatial Contributions (SDSC), and Proton Net Charges *q*_H_

proton	core	SSSC	SDSC	total	δ_cal_	δ_Expt_^1^	*q*_H_
H_a_	18.85	8.91	–3.41	24.35	7.14	6.99	0.1675
H_b_	19.83	6.95	–1.74	25.04	6.44	6.43	0.1320
H_c_	19.76	5.79	–1.70	23.86	7.62	7.60	0.1387
H_d_	19.79	5.49	–1.88	23.41	8.08	8.04	0.1385
H_e_	19.76	5.36	–1.88	23.24	8.24	8.18	0.1398
H_f_	19.78	5.25	–1.76	23.27	8.22	8.16	0.1433

Looking at the core contributions, it can be observed that protons c, d, e, and f show almost the same value (maximum deviation 0.03 ppm), indicating a very similar internal region. The same protons display a decreasing sum of shielding spatial contributions (SSSC) going from H_c_ to H_f_ (Δσ = 0.54 ppm) in parallel with a decreasing sum of deshielding spatial contributions (SDSC); H_f_ deviates a little from this tendency. This behavior changes a lot for H_a_ and H_b_. The latter shows an SSSC equal to 6.95 ppm, which surpasses by 1.16 ppm the value relative to H_c_, accounting quantitatively for the δ_Expt_(H_c_) – δ_Expt_(H_b_) = 1.17 ppm. This increment of the SSSC can be traced back to the somewhat larger shielding contributions from the carbon atoms of the lower benzene rings and from the K-regions of the coronene subunit, visible on the right in the top row in Figure 5. With regard to H_a_ everything is changed: the core contribution is the smallest, while in absolute value both the SSSC and the SDSC are the largest. This leads to cross compensations, which collocates the H_a_ chemical shift midway between H_b_ and H_c_. Looking at Figure 5, top row on the left, it is possible to locate the source of the largest deshielding on the carbon atoms forming the fjord and the source of the largest shielding on the carbons in the lower benzene rings and K-regions as well. In other words, seen along the *x*-axis, H_a_ is inside two counterrotating currents and undergoes their opposite effects, i.e., the paratropic one giving a deshielding effect and the diatropic one giving a shielding effect. The smaller core contribution can be attributed to a loss of electron charge in the intimate region around the nucleus, which is compatible with the calculated Mulliken populations, showing that the four H_a_’s have the highest positive charge within the set of hydrogen atoms. It is known that, upon formation of CH−π interactions, the hydrogen loses electrons.^35^ In our case (Figure 3) there is no bond path between the hydrogen and carbon atoms of the above ring. However, the C–C bond path is curved toward the hydrogen, indicating that the hydrogen is involved in this interaction.

## Conclusions

Returning to the unsolved questions underlined before for infinitene, we have shown the following: (iv) As for the strength of the current, induced by a perturbing magnetic field perpendicular to the central C–C bonds of the two stacked naphthalene subunits, in the K-regions it is 1.5 times weaker than the diatropic current that circulates on pristine coronene rim, while in the fjord the current is 2 times stronger than the paratropic current on pristine coronene hub. (v) The exaltation of diamagnetism is fairly low, being only 73% of the expected value. (vi) The high-field ^1^H NMR signals are due to the global currents flowing on the fjord region and on the carbon atoms on the lower benzene rings and K-edges, with a fairly large deshielding effect on H_a_, the former, and shielding effects on both H_a_ and H_b_, the latter.
